# Unexpected Response to Hormonal Therapy in Metastatic Neuroendocrine-Differentiated Prostate Adenocarcinoma: A Case Report

**DOI:** 10.7759/cureus.91967

**Published:** 2025-09-10

**Authors:** Kaoutar Maadin, Mehdi Alem, Zineb Benbrahim, Samia Arifi, Nawfel Mellas

**Affiliations:** 1 Department of Medical Oncology, Faculty of Medicine, Pharmacy and Dental Medicine of Fez, University Sidi Mohamed Ben Abdellah, Hassan II University Hospital Center, Fez, MAR

**Keywords:** androgen deprivation therapy, hepatic metastases, hormone sensitivity, neuroendocrine differentiation, prostate cancer, visceral crisis

## Abstract

Prostate cancer with neuroendocrine differentiation (NEPC) is an aggressive subtype typically considered androgen-indifferent and primarily managed with chemotherapy. Presentation as a hepatic visceral crisis constitutes a medical emergency with high mortality, often requiring urgent systemic chemotherapy. We report the case of a 52-year-old male patient who presented with life-threatening hepatic visceral crisis as the initial manifestation of metastatic neuroendocrine differentiated prostate adenocarcinoma, Gleason score 9 (4 + 5), with a prostate-specific antigen (PSA) level of 137 ng/mL. Remarkably, first-line androgen deprivation therapy (ADT) as monotherapy led to rapid and marked clinical improvement, with complete normalization of liver function tests within one month, prior to any chemotherapy. The patient subsequently underwent consolidation chemotherapy and remains in complete and durable remission, with an undetectable PSA level. This case illustrates that significant sensitivity to ADT can persist even in the context of aggressive prostate cancer with neuroendocrine features and acute organ dysfunction. It challenges the routine exclusion of hormonal therapy in such scenarios and highlights the potential of ADT as a life-saving therapeutic bridge in critical clinical settings.

## Introduction

Prostate adenocarcinoma (PCa) is typically an androgen-dependent malignancy, and androgen deprivation therapy (ADT) remains the standard of care for metastatic disease. However, most tumors eventually progress to castration-resistant prostate cancer (CRPC).

High-grade adenocarcinoma with focal neuroendocrine differentiation is a rare and aggressive variant characterized by heterogeneous biology, often associated with low or absent prostate-specific antigen (PSA) expression, visceral metastases, and partial androgen receptor (AR) independence. In such cases, platinum-based chemotherapy is generally considered the preferred treatment, as hormonal therapy is expected to show limited efficacy.

The clinical course of these aggressive forms can be further complicated by visceral crisis, defined as acute and severe organ dysfunction resulting from rapidly progressive metastatic disease. Hepatic visceral crisis, in particular, represents a medical emergency with high early mortality, frequently requiring the urgent initiation of systemic chemotherapy to avert fatal outcomes.

Herein, we report the case of a patient diagnosed with high-grade PCa with focal neuroendocrine differentiation, who presented with an imminent, life-threatening hepatic visceral crisis. Unexpectedly, the patient achieved a dramatic remission following ADT alone, prior to the initiation of chemotherapy. This observation challenges the conventional perception of universal hormone resistance in aggressive prostate cancer variants and highlights that hormonal therapy may still provide clinically meaningful benefit in select, critical scenarios.

## Case presentation

A 52-year-old male patient with no significant past medical, surgical, or family history of cancer presented with a three-month history of dyspepsia and irritative lower urinary tract symptoms, including urinary frequency and dysuria. He denied abdominal pain, gastrointestinal bleeding, or hematuria. On clinical examination, his Eastern Cooperative Oncology Group (ECOG) performance status was 1. The abdomen was soft and non-tender but revealed a significant hepatomegaly, with a liver span of 20 cm.

**Figure 1:**
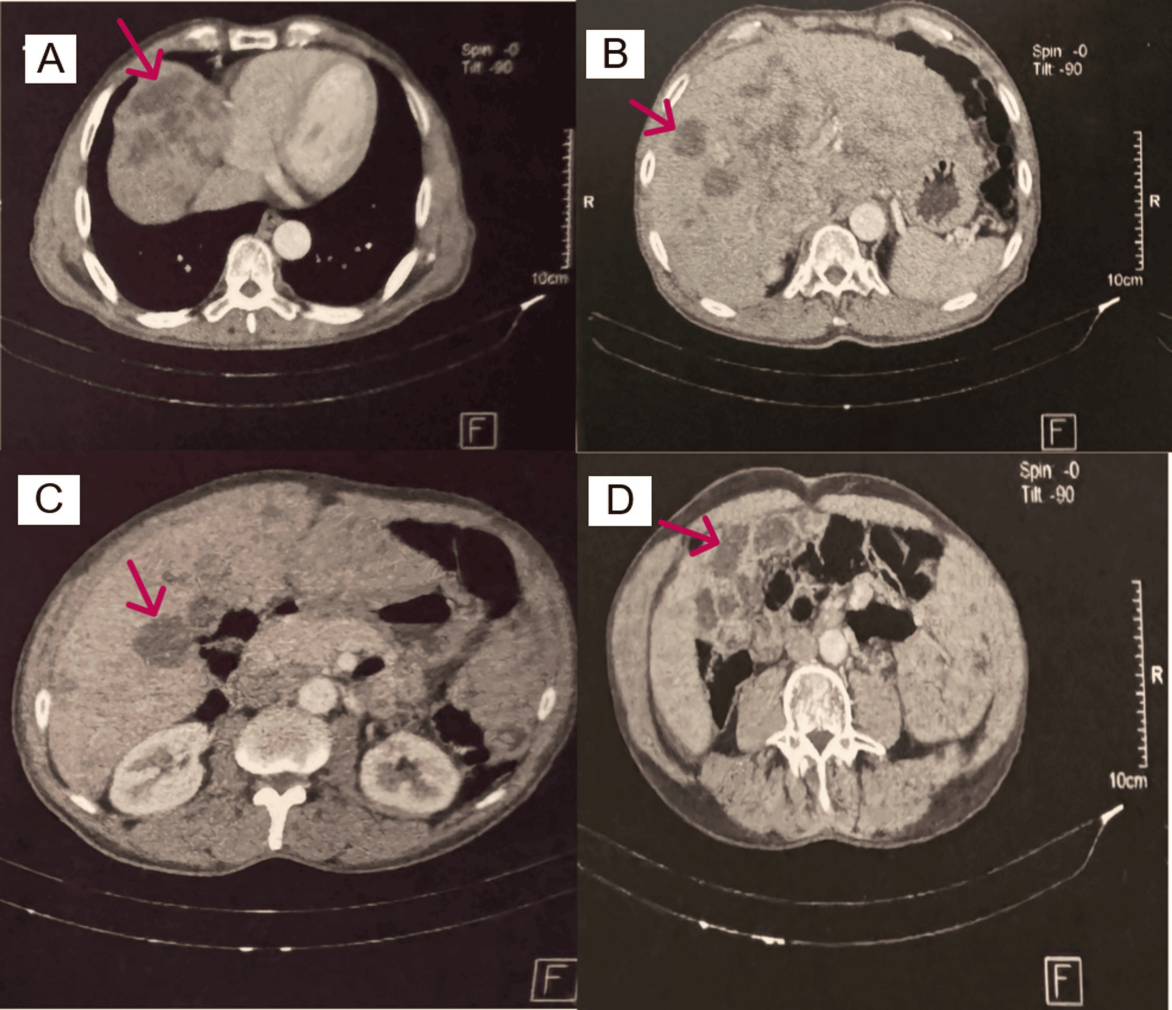
Extensive hepatic metastases from prostate adenocarcinoma with neuroendocrine differentiation associated with hepatic visceral crisis on abdominal CT (A) Large confluent hypodense mass (red arrow) in the right hepatic lobe, measuring approximately 154 mm. (B) Multiple irregular hypodense nodules involving both hepatic lobes (red arrow). (C) Diffuse metastatic infiltration causing distortion of the normal liver architecture (red arrow). (D) Heterogeneous hepatic parenchyma consistent with advanced metastatic disease (red arrow). CT: computed tomography

**Figure 2:**
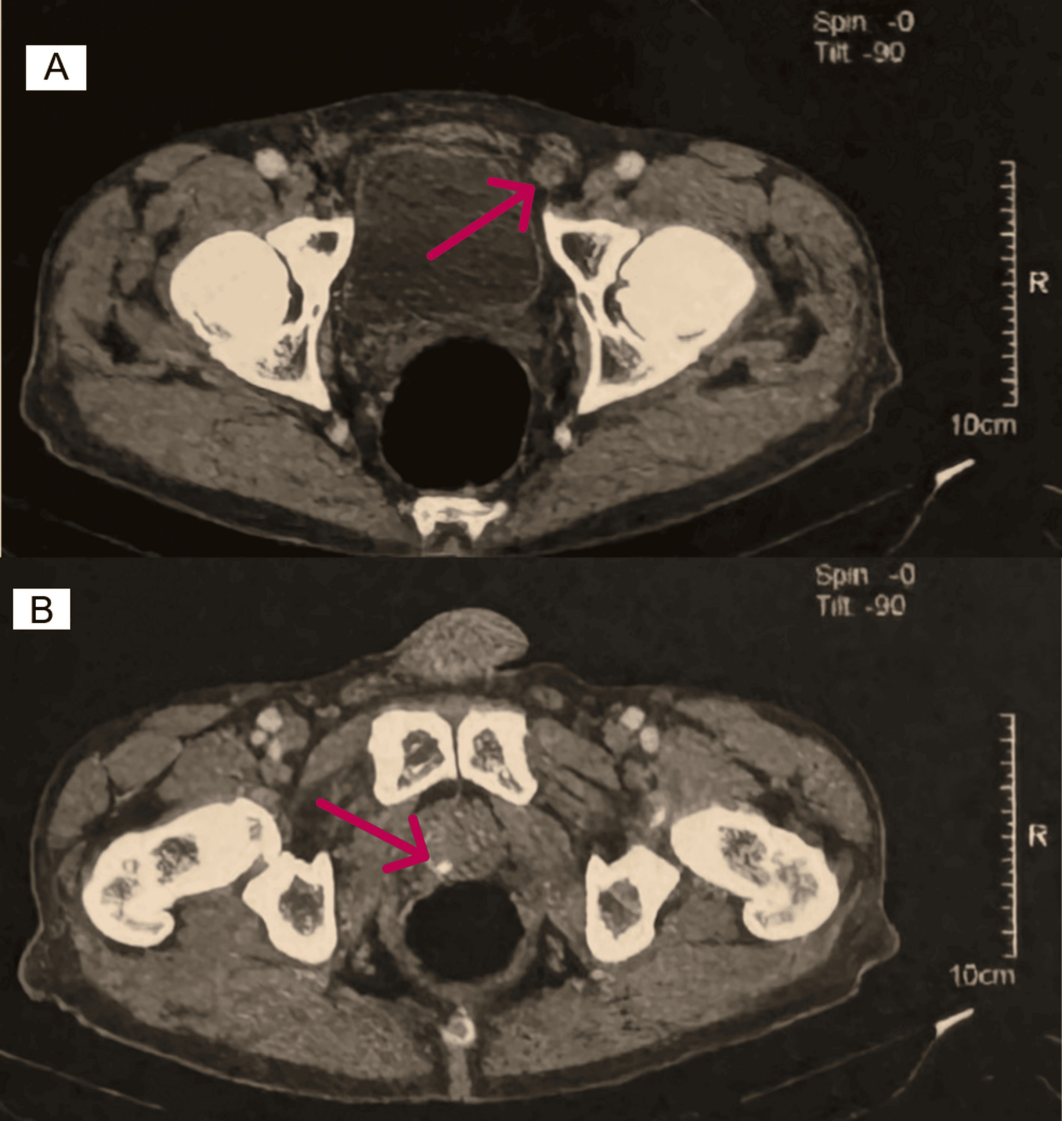
Pelvic CT showing right iliac lymphadenopathy and prostatic lesion (A) Axial CT showing a 29 mm right iliac lymphadenopathy (red arrow). (B) Axial CT demonstrating a 13 mm enhancing lesion (red arrow) in the right peripheral zone of a normal-sized prostate, consistent with the primary tumor. CT: computed tomography

Initial diagnostic workup included a thoraco-abdomino-pelvic computed tomography (CT) scan, which demonstrated a massive metastatic burden in a diffusely enlarged liver, the largest confluent lesion measuring 154 mm in diameter (Figure 1). The scan also revealed right iliac lymphadenopathy (29 mm) and a 13 mm enhancing lesion in the right peripheral zone of a normal-sized prostate (Figure 2). Gastroscopy and colonoscopy were unremarkable, and bone scintigraphy revealed multiple secondary bone lesions.

Laboratory investigations showed severe hepatic cytolysis and cholestasis, confirming a hepatic visceral crisis: aspartate aminotransferase (AST) 240 U/L, alanine aminotransferase (ALT) 136 U/L, gamma-glutamyl transferase (GGT) 677 U/L, and total bilirubin 14 mg/dL. Tumor markers were significantly elevated, with PSA at 137 ng/mL, carcinoembryonic antigen (CEA) at 897 ng/mL, and carbohydrate antigen 19-9 (CA 19-9) at 52.4 U/mL. Laboratory results are summarized in Table 1. The markedly elevated PSA level was consistent with an adenocarcinoma biology, while the very high CEA suggested aggressive disease behavior with focal neuroendocrine differentiation.

**Table 1:** Summary of initial laboratory results

Parameter	Result	Reference range
Aspartate aminotransferase (AST/GOT)	240 U/L	≤41 U/L
Alanine aminotransferase (ALT/GPT)	136 U/L	≤42 U/L
Gamma-glutamyl transferase (GGT)	677 U/L	0-55 U/L
Total bilirubin	14.00 mg/dL	0.1-1.2 mg/dL
Conjugated bilirubin	8.00 mg/dL	0.0-0.3 mg/dL
Unconjugated bilirubin	6.00 mg/dL	0.1-1.0 mg/dL
Alkaline phosphatase	485 U/L	0-105 U/L
Carcinoembryonic antigen (CEA)	897.00 ng/mL	<10 ng/mL
Carbohydrate antigen 19-9 (CA 19-9)	52.40 U/mL	<37 U/mL
Prostate-specific antigen (PSA)	137 ng/mL	<4 ng/mL

A core biopsy of a hepatic lesion revealed a poorly differentiated adenocarcinoma with focal expression of CK7 and MUC1. Given the low and focal expression of PSA, a prostatic origin was suspected. A subsequent transrectal prostate biopsy confirmed a diagnosis of high-grade prostatic adenocarcinoma (Gleason score 9 (4 + 5), International Society of Urological Pathology (ISUP) Grade Group 5) with focal neuroendocrine differentiation, based on immunohistochemical staining positive for chromogranin and synaptophysin. A more comprehensive immunohistochemical panel (AR, Ki-67, and NKX3.1), genomic profiling (TP53, RB1, PTEN, and MYCN), and dual-tracer imaging (prostate-specific membrane antigen (PSMA)-PET and fludeoxyglucose (FDG)-PET) were not available in our setting, which we acknowledge as limitations.

The patient was planned for combination therapy. ADT was initiated using a gonadotropin-releasing hormone (GnRH) agonist and bicalutamide as an anti-androgen. Remarkably, within the first month of ADT alone and prior to the initiation of chemotherapy, the patient showed dramatic clinical improvement, with complete normalization of liver function tests. Given the neuroendocrine features and the aggressive clinical presentation, the choice of adding carboplatin to docetaxel was based on extrapolation from data on aggressive variant prostate cancer. The patient subsequently completed six cycles of docetaxel plus carboplatin. At the most recent follow-up, he remains asymptomatic, with an undetectable PSA level (0.01 ng/mL) and no radiological evidence of recurrence.

## Discussion

We report a rare case of a treatment-naïve 52-year-old man presenting with high-grade (Gleason 9 (4 + 5)) prostatic adenocarcinoma with focal neuroendocrine differentiation, revealed by a life-threatening hepatic visceral crisis. Despite clinical and pathological features suggestive of an aggressive phenotype, including immunohistochemical evidence of focal neuroendocrine differentiation and a massive metastatic burden, the patient experienced a rapid and profound remission following ADT alone. This unexpected outcome challenges conventional therapeutic paradigms for such advanced presentations and underscores the biological heterogeneity of aggressive prostate cancer.

High-grade adenocarcinoma with focal neuroendocrine differentiation represents a recognized mechanism of therapeutic resistance and disease progression. This transdifferentiation is often driven by concurrent loss of tumor suppressor genes such as TP53 and RB1, promoting lineage plasticity and the expression of neuroendocrine-associated transcription factors including ASCL1 and NEUROD1 [1,2]. The resulting tumors typically exhibit reduced AR signaling, rendering ADT less effective. In such cases, visceral crisis-particularly hepatic-represents a medical emergency with high early mortality, generally prompting the immediate initiation of platinum-based chemotherapy, a strategy extrapolated from the management of small-cell lung cancer [3,4]. In contrast, our patient’s dramatic response to ADT alone illustrates a paradoxical but clinically meaningful therapeutic window.

The most plausible explanation lies in intratumoral heterogeneity. The diagnosis of a high-grade adenocarcinoma with focal neuroendocrine differentiation suggests the coexistence of multiple tumor subclones. It is likely that the dominant proliferative clone responsible for hepatic metastases retained androgen sensitivity and that ADT induced apoptosis within this population, leading to rapid stabilization [5]. The neuroendocrine component, although histologically evident and contributory to aggressiveness, may have represented a minor, non-dominant subclone at the time. Thus, detection of neuroendocrine markers on a biopsy should not automatically imply universal hormone resistance throughout the tumor mass [6].

This interpretation is further supported by the biomarker profile. The PSA level of 137 ng/mL is atypically high for pure neuroendocrine prostate cancer and supports the predominance of an AR-driven adenocarcinoma clone [7]. Conversely, the markedly elevated CEA at 897 ng/mL, while non-specific, is frequently associated with poorly differentiated tumors and neuroendocrine differentiation, reinforcing the concept of a mixed histologic phenotype [5]. Ideally, this case would have benefitted from dual-tracer PET imaging (PSMA-PET and FDG-PET) to characterize tumor heterogeneity in vivo [8,9].

From a clinical perspective, this case contributes to discussions about the optimal strategy for de novo high-risk metastatic prostate cancer. Recent trials such as PEACE-1 and ARASENS have established triplet therapy (ADT + a novel hormonal agent such as abiraterone + docetaxel) as the standard of care [10,11]. However, in the precarious context of a visceral crisis, our case suggests that a sequential therapeutic approach starting with ADT as a low-toxicity bridge may be advantageous. ADT alone reversed life-threatening hepatic dysfunction, improved performance status, and enabled subsequent chemotherapy, thereby avoiding early cytotoxic treatment in a critically ill patient.

Naturally, this report is limited by its nature as a single case. Confirmation of our heterogeneity hypothesis would ideally have required serial biopsies to monitor histological evolution after ADT, as well as complementary investigations such as an extended immunohistochemical panel, genomic profiling, or dual-tracer PET imaging. While these data were not available in our setting, their absence does not undermine the diagnosis of high-grade adenocarcinoma with focal neuroendocrine differentiation and rather reflects the real-world challenges encountered in resource-constrained environments.

## Conclusions

This case highlights a paradoxical clinical scenario: a high-grade PCa with focal neuroendocrine differentiation presenting as a life-threatening hepatic visceral crisis. The dramatic and rapid response to ADT alone prior to the initiation of chemotherapy challenges the prevailing dogma that considers such tumors inherently hormone-resistant. This observation underscores the critical role of tumor heterogeneity, suggesting that even in aggressive histological variants, a dominant hormone-sensitive clone may dictate the acute clinical trajectory. Nevertheless, this conclusion arises from a single case, and caution is required in extrapolating these findings. Further reports and studies are needed to better define the role of ADT in similar presentations.

## References

[1] Ku SY, Rosario S, Wang Y (2017). Rb1 and Trp53 cooperate to suppress prostate cancer lineage plasticity, metastasis, and antiandrogen resistance. Science.

[2] Sinha S, Nyquist MD, Corella A, Coleman I, Nelson PS (2018). Abstract LB-199: role of ASCL1 in neuroendocrine prostate cancer progression. Cancer Res.

[3] Cornford P, van den Bergh RC, Briers E (2021). EAU-EANM-ESTRO-ESUR-SIOG guidelines on prostate cancer. Part II-2020 update: treatment of relapsing and metastatic prostate cancer. Eur Urol.

[4] Flauto F, Neola G, Caso C (2025). Evaluating treatment efficacy in metastatic hormone-sensitive prostate cancer patients with visceral disease: a systematic review and network meta-analysis. Eur Urol Oncol.

[5] Yamada Y, Beltran H (2021). Clinical and biological features of neuroendocrine prostate cancer. Curr Oncol Rep.

[6] Nelson EC, Cambio AJ, Yang JC, Ok JH, Lara PN Jr, Evans CP (2007). Clinical implications of neuroendocrine differentiation in prostate cancer. Prostate Cancer Prostatic Dis.

[7] Kouroukli O, Bravou V, Giannitsas K, Tzelepi V (2024). Tissue-based diagnostic biomarkers of aggressive variant prostate cancer: a narrative review. Cancers (Basel).

[8] Hofman MS, Emmett L, Sandhu S (2021). [177Lu]Lu-PSMA-617 versus cabazitaxel in patients with metastatic castration-resistant prostate cancer (TheraP): a randomised, open-label, phase 2 trial. Lancet.

[9] Spratt DE, Gavane S, Tarlinton L, Fareedy SB, Doran MG, Zelefsky MJ, Osborne JR (2014). Utility of FDG-PET in clinical neuroendocrine prostate cancer. Prostate.

[10] Fizazi K, Tran N, Fein L (2017). Abiraterone plus prednisone in metastatic, castration-sensitive prostate cancer. N Engl J Med.

[11] Smith MR, Hussain M, Saad F (2022). Darolutamide and survival in metastatic, hormone-sensitive prostate cancer. N Engl J Med.

